# 5-Nitro-2,4-Dichloropyrimidine as an Universal Model for Low-Energy Electron Processes Relevant for Radiosensitization

**DOI:** 10.3390/ijms21218173

**Published:** 2020-10-31

**Authors:** Thomas F. M. Luxford, Stanislav A. Pshenichnyuk, Nail L. Asfandiarov, Tomáš Perečko, Martin Falk, Jaroslav Kočišek

**Affiliations:** 1 J. Heyrovský Institute of Physical Chemistry of the Czech Academy of Sciences, Dolejškova 3, 18223 Prague, Czech Republic; thomas.luxford@jh-inst.cas.cz; 2 Institute of Molecule and Crystal Physics UFRC RAS, October Avenue 151, 450075 Ufa, Russia; nail_asf@mail.ru; 3 Institute of Biophysics of the Czech Academy of Sciences, Kralovopolska 135, 612 65 Brno, Czech Republic; tomas.perecko@ibp.cz

**Keywords:** low-energy electrons, dissociative electron attachment, pyrimidine, radiosensitizer

## Abstract

We report experimental results of low-energy electron interactions with 5-nitro-2,4-dichloropyrimidine isolated in the gas phase and hydrated in a cluster environment. The molecule exhibits a very rare combination of many so far hypothesized low-energy electron induced mechanisms, which may be responsible for synergism in concurrent chemo-radiation therapy of cancer. In contrast to many previous efforts to design an ideal radiosensitizer based on one mode of action, the present model molecule presents an alternative approach, where several modes of action are combined. With respect to the processes induced by the low-energy electrons, this is not a trivial task because of strong bond specificity of the dissociative electron attachment reaction, as it is discussed in the present paper. Unfortunately, low solubility and high toxicity of the molecule, as obtained from preliminary MTT assay tests, do not enable further studies of its activity in real biological systems but it can advantageously serve as a model or a base for rational design of radiosensitizers.

## 1. Introduction

Radiation therapies combined with chemotherapy often exhibit synergistic effects. The synergy may be caused by increased radiation damage, inhibition of DNA repair, cell-cycle synchronization, increased cytotoxicity against hypoxic cells, inhibition of prosurvival pathways or other physical, chemical or biochemical mechanisms [1]. Except for common chemo-radio therapeutics such as cisplatin, several other molecules used or proposed as radiosensitizers are containing functional groups with high electron affinity. Examples are halogen-, thio-, azido- or cyano-substituted DNA bases [2,3,4,5], bromopyruvic acid [6], nitroimidazoles [7,8,9] or halogenated nucleosides [10,11]. This fact, together with well known action of secondary low-energy electrons during the radiation interaction with living matter [12,13,14], induced significant efforts to explore the role of secondary electrons in the radiosensitization (see, e.g., recent reviews [15,16,17,18]). Several mechanisms have been proposed, which may be important and may be even used to rationally design new radiosensitizers and chemo-radiotherapy drugs. The main processes proposed so far, which may be based on the action of secondary low-energy electrons, are:

1. DEA—dissociaitive electron attachment AB + e^−^ → A + B^−^,

which is the most intensely studied. The main reason for increased interest in DEA is its ability to break bonds at sub-excitation energies of interacting electrons, or even by interaction with already solvated electrons [19]. DEA can induce formation of reactive radicals such as OH${}^{•}$, which can increase the DNA damage after irradiation [20,21] or NO acting on several levels of biological hierarchy from chemical changes on molecular level to physiological changes on the tissue level [22,23,24]. DEA also produces anions such as Cl${}^{-}$ [25] or DNA base radicals and anions, which may be incorporated into the DNA structure, cross-link or influence other biological processes [26,27,28,29].

2. AEA—asociative electron attachment AB + e^−^ → AB^−^

has gained more interest in recent years. AEA results in the formation of long lived molecular anions. Such anions may be better transferred to the vicinity of DNA in comparison to their neutral precursors [30]. The large electron affinity of these compounds results in excess energy of the anion with respect to its neutral precursor, which may be transferred to the surrounding environment [31]. Such energy transfer may contribute to the total linear energy transfer of the high energy projectile or enhanced local heating with physical as well as chemical or biological consequences [32].

3. DNA sensitization

Several other mechanisms were proposed, where the secondary low-energy electrons do not interact with molecules directly, but only after its chemical modification or binding to the target—typically DNA. Organometallics are the class of molecules which have been most studied in this manner. [33,34]. Proposed actions include electron transfer [28,35,36], enhanced production of secondary electrons [37] or sensitization of DNA to secondary electrons [38,39,40,41].

**Figure 1:**
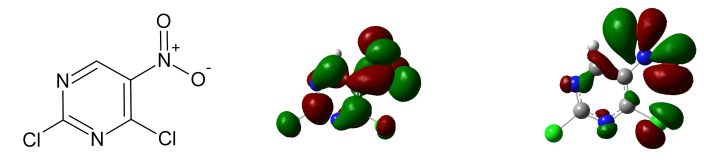
Sketch of neutral 5-nitro-2,4-dichloropyrimidine structure (**left**), its Lowest unoccupied orbital (**center**), and Highest occupied orbital of anion (**right**) calculated at DFT B3LYP/6-31G+(d) level of theory in Gaussian [42].

The previously mentioned fundamental studies then often propose rational design of novel radiosensitizers on the particular studied process. In the present study we reversed the approach and applied the above mentioned knowledge together with our experience studying low-energy electron induced processes to nitro-, halo-substituted and biologically relevant molecules to propose a simple model molecule that covers several of these mechanisms. The purpose of this study is not to propose a new radiosensitizer, as such a process requires the inclusion of many parameters out of field of our expertise. Our goal was to demonstrate that with respect to low-energy electrons, several modes of action can be effectively combined on a single small molecule. Pre-screening of several pyrimidines and purines in the gas phase resulted in the selection of 5-nitro-2,4-dichloropyrimidine (C${}_{4}$HCl${}_{2}$N${}_{3}$O${}_{2}$, Figure 1) for further studies. The interaction of the isolated and hydrated molecule with free low-energy electrons in vacuum was studied on two experimental setups, gaining information about anion lifetime, electron affinity and fragmentation reactions induced by low-energy electrons. This way, we provide experimental evidence that in the case of 5-nitro-2,4-dichloropyrimidine all the above mentioned processes relevant for radiosensitization by electron affinic molecules are possible. Or otherwise, if any of the so far proposed hypotheses about the action of low-energy electrons is correct then 5-nitro-2,4-dichloropyrimidine should exhibit a radiosensitizing effect.

## 2. Materials and Methods

### 2.1. Electron Attachment to Isolated 5-Nitro-2,4-Dichloropyrimidine

Anion yields after electron attachment to isolated 5-nitro-2,4-dichloropyrimidine and lifetime of its molecular negative ion in respect of electron detachment were evaluated on a sector instrument in Ufa [43]. The sample molecule (Sigma Aldrich, 97% purity) was sublimed at 450 K into the collision cell where it was irradiated by a magnetically guided electron beam. The electron energy was varied in the 0–14 eV range. The energy scale was calibrated by SF${}_{6}^{-}$/SF${}_{6}$ 0 eV resonance. The ions formed in electron-molecule collisions were extracted towards the magnetic sector mass spectrometer and electron energy dependent anion yields were obtained for a particular mass-to-charge ratio. Evaluation of the mean electron autodetachment time was based on the detection of fast neutral species in the field-free region between the mass analyzer and the secondary electron multiplier. These fast neutrals are formed by electron detachment from negative ions accelerated in the sector part of the instrument and their yield allows for estimation of the autodetachment time [44].

### 2.2. Electron Attachment to Dry and Microhydrated 5-Nitro-2,4-Dichloropyrimidine in Molecular Beam

Free electron attachment to clusters of microhydrated 5-nitro-2,4-dichloropyrimidine was studied on the CLUB (ClUster Beam) experimental setup in arrangement identical to that described in [45]. 5-nitro-2,4-nichloropyrimidine molecule (Sigma Aldrich, 97% purity) was sublimed in a resistively heated reservoir and mixed with pure He buffer gas or with buffer gas (He or Ne) with admixture of water provided by an in line ESI Pergo gas humidifier. The mixture was then expanded through divergent nozzle into vacuum at stagnation pressures 1-2 bar as specified for individual spectra in the results section. This way a beam of cold isolated molecules or microhydrated clusters was prepared that was crossed by magnetically collimated beam of low-energy electrons. The elctrons were prepared in a simple gun consisting of a tungsten cathode emitter and set of three electrodes. Formed anions were analyzed by reflectron time-of-flight (TOF) spectrometer obtaining 3D spectra of anion yield dependence on electron energy and mass to charge ratio.

### 2.3. Cytotoxicity of 5-Nitro-2,4-Dichloropyrimidine

#### 2.3.1. Cell Lines and Culturing

Human squamous cell carcinoma cell line FaDu (referred to as FaDu; ATCC®HTB-43^TM^, USA) was cultured in RPMI1640 medium supplemented with 10% low endotoxin fetal bovine serum FBS and 1% Penicillin/Streptomycin (GIBCO). Human Gingival Fibroblasts (referred to as hGF; 300703, CLS Cell Lines Service, Germany) were cultured in DMEM:Ham’s F12 medium (GIBCO), supplemented with 10% FBS (GIBCO) and 1% Penicillin/Streptomycin (GIBCO). Both cell types were held under standard conditions, i.e., at $37{\;}^{\circ }$C and a humidified atmosphere containing 5% CO${}_{2}$. In all experiments, asynchronously and exponentially growing cells were used.

#### 2.3.2. MTT Assay

Cells were seeded at amounts of 3000 (FaDu) or 5000 (hGF) cells per well in 96-well plates. After 24 h of culturing, when the cells grew exponentially, the culturing medium was replaced with a medium containing increasing concentrations of 5-nitro-2,4-dichloropyrimidine, ranging from 5 μM to 250 μM for individual samples. The samples with corresponding concentrations (0.005–0.25%) of DMSO served as controls to test possible effects of this solvent on the cell viability. Treated cells were incubated for 48 h before adding 25 μL of 2.5 mg/mL MTT (3-(4,5-Dimethylthiazol-2-yl)-2,5-Diphenyltetrazolium Bromide) per 100 μL of the culturing medium. After 2 h incubation ($37{\;}^{\circ }$C, 5% CO${}_{2}$), the medium was aspirated and the cells were lysed with 150 μL of 10% Triton/HCl. The absorbance was read at 570 nm using a microplate spectrophotometer Infinite M200 Pro (Tecan, Austria). The measured values were normalized to corresponding DMSO controls. For DMSO, the values were normalized to non-treated control. The results are plotted as the mean ± SEM values of 3–4 replicates performed in triplicates.

## 3. Results and Discussion

In the present study we performed electron attachment experiments on two different experimental setups. First, we performed electron affinity and anion lifetime measurements in the gas phase using the sector instrument in Ufa. The instrument enabled also precise determination of individual resonances resulting in the formation of particular anions. These gas phase measurements were then complemented by measurements of anion MS at different hydration conditions at the CLUB setup in Prague. This way, we get detailed information about anion nature as well as its behavior upon solvation.

### 3.1. Parent Anion

The anion lifetimes are measured using negative ion lifetime mass spectrometry [46]. Results for the M${}^{-}$ anion detachment show a ∼1.2 ms long lifetime of the anion. This value is a unique feature of the present molecule, as a typical lifetime of halogen- or nitro- containing aromatic compounds are in the range of hundreds of microseconds [47]. An anion with such a long lifetime may act by two of the mechanisms mentioned in the introduction. First as an antennae after binding to the DNA structure [48]. For such binding the molecule has to be compatible with the DNA structure, such as pyrimidine, and may undergo proper solution chemistry, which will be discussed further. Second, it may be better transported to the vicinity of the DNA or other molecular sites, where it can act by its different modes of action [30]. From this point of view the present anion lifetime is unique and may overcome many of the so far used radiosensitizers.

The anion lifetimes enable us to calculate the adiabatic electron affinity of the molecule. The gas phase adiabatic electron affinity based on the experimental data is 2.92 eV and calculated gas and liquid phase electron affinities are 2.11 eV and 3.58 eV, respectively. Similar to lifetime, also the electron affinity value is unusually high [49], it is more than twice as high as similar pure nitro-compounds [47]). The large electron affinity may result in the enhancement of the linear energy transfer (LET) to the tissue as described in the work of Postulka [31].

### 3.2. Fragment Anions

**Figure 2:**
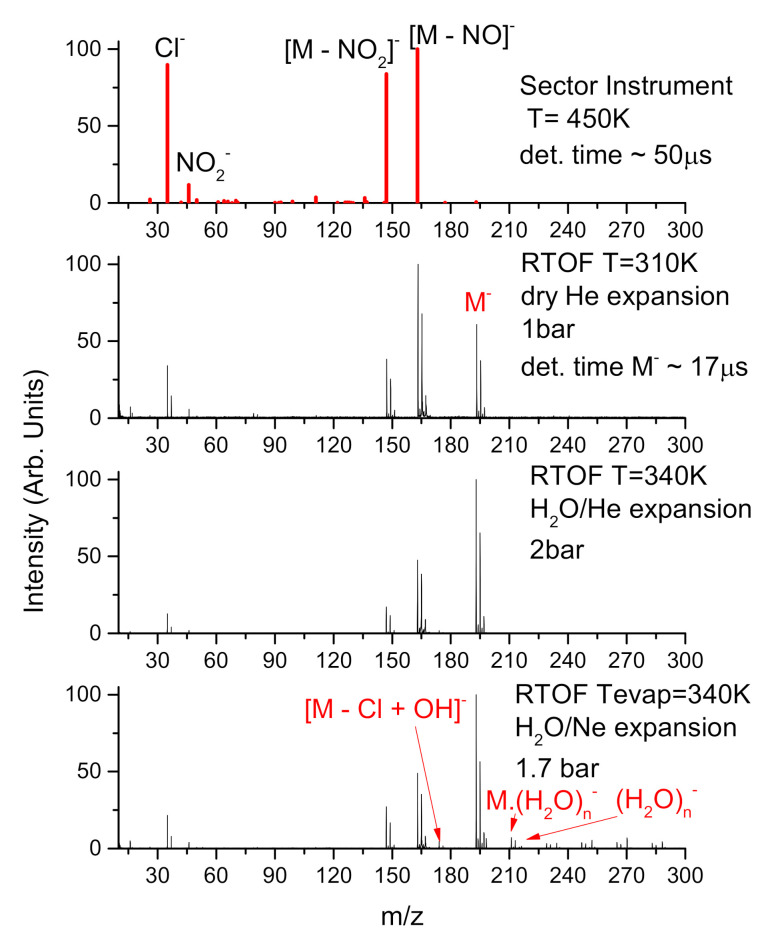
Mass spectra of negative ions formed by C${}_{4}$HCl${}_{2}$N${}_{3}$O${}_{2}$ interaction with electrons in the range of 0–14 eV. Top bar graph spectrum is for isolated molecule, obtained as an integral of the individual ion yields measured at the electron attachment spectrometer with sector mass analyzer in Ufa. Below are the spectra from RTOF measurements with molecular beam of C${}_{4}$HCl${}_{2}$N${}_{3}$O${}_{2}$ in He, hydrated He and hydrated Ne as a buffer gas—increasing hydration from top to bottom.

Figure 2 shows the anion mass spectra, which are obtained as a sum of spectra taken at individual energies from 0 eV to 14 eV to cover all resonance channels. The individual DEA fragmentation channels are assigned in the Table 1 together with calculated thermodynamic thresholds of the reactions. The top spectrum in Figure 2 is obtained using electron attachment spectrometer equipped with sector mass analyzer in Ufa and characterizes decomposition of the isolated molecule which was evaporated at high temperature of 450 K to measure detailed ion yields for all fragmentation channels. The second spectrum from top was taken with reflectron time-of-flight (RTOF) instrument in Prague and it is also for the isolated molecule, but taken at low sublimation temperature of 310 K and in the molecular beam expansion with helium that further cools the neutral precursor.

**Figure 3:**
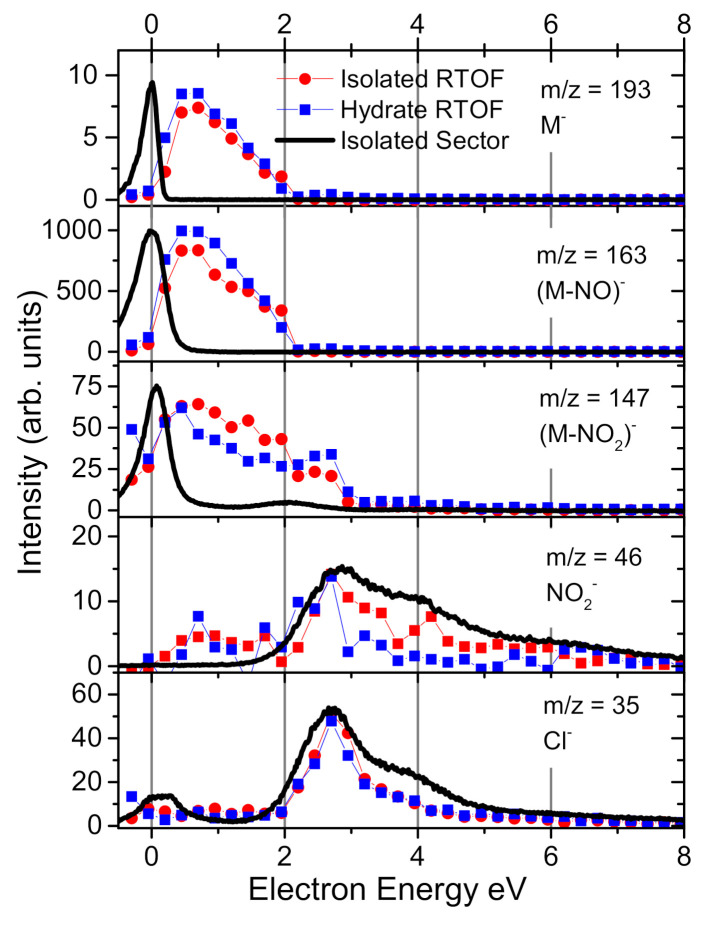
Electron energy dependent ion yelds for 5 most intense individual reaction channels of EA to 5-nitro-2,4-dichloropyrimidine as identified from measurements of isolated molecule in molecular beam using RTOF.

There is a clear difference between the spectra of isolated molecules measured using the different instruments. The main difference is in the intensity of the parent anion signal, which is much more pronounced in the beam spectrum. This can have several reasons. (i) different timescale for the anion detection in the present experiments ∼50 μs and 17 μs for sector and RTOF instrument, respectively. However, due to the long anion lifetime, the effect of different detection times should be low, less than 10%. (ii) different integration region in the electron energy dimension. A simple electron gun is used in Prague experiment, with significant decrease of the electron current below ∼1 eV (see [50] for detailed discussion). Therefore signals for anions formed at low energies should be supressed in RTOF experiment. We can see this effect also from Figure 3, which shows the ion yields in different experiments as a function of electron energy. As the parent anion resonance lies at the lowest energies in the spectrum the Ufa experiment with sector instrument should be much more sensitive to parent anions. Our observed effect is the opposite and therefore the only explanation remains (iii) the different temperature of anions in the two experiments. The high anion temperature in the Ufa experiment results in higher fragmentation of the anions formed after electron attachment. The top two panels of Figure 2 therefore show fragmentation patterns after electron attachment at two extremes—very hot molecule (sector) and very cold molecule (RTOF).

In the CLUB experiment, the molecule can be very cold, reducing the fragmentation. Still, several dissociation channels remain opened. The main fragments are (M-NO${}_{2}$)${}^{-}$ and (M-NO)${}^{-}$. Therefore, NO${}_{x}$ species may be effectively formed in combination with radiation. These radicals may enhance the radiation damage by several mechanisms [22,24]. The halo-substitution causes that radicals formed after NO and NO${}_{2}$ dissociation are electronegative. These radical anions may incorporate into DNA or other important biological targets and cause electron transfer [51,52] to the target or its further sensitization to ionizing radiation [26,53].

Except for the neutral radicals NO and NO${}_{2}$ we can also see formation of reactive anions such as NO${}_{2}^{-}$ or Cl${}_{2}^{-}$. These can again cause chemical as well as biological changes in the tissue.

Several of the formed anions have high electron affinities exceeding 3 eV (see Table 2), which is a common feature for electrophilic radiosensitizer used in clinical practice. It is also important that the Cl loss channel is endothermic (see Table 1). Therefore, the molecule does not undergo rapid Cl loss by dissolution or DEA as it is common for many other halocarbons and stable molecular anion can be formed as discussed above.

### 3.3. Effect of Microhydration

An important parameter influencing the DEA reaction is the environment surrounding the molecule. The DEA reaction may be significantly altered in solution as shown in the previous studies with secondary electrons created by ionizing radiation in bulk [56,57]. The environmental effects on DEA may be then well identified in cluster studies with free low-energy electrons [45,58,59], as they will be discussed in the following text.

The bottom graphs in Figure 2 shows the fragmentation data after low-energy electron interaction with microhydrated C${}_{4}$HCl${}_{2}$N${}_{3}$O${}_{2}$. We can see suppression of the fragmentation channels similar to other so far studied molecules (see, e.g., [31,45]). However, we do not observe complete closings of the channels. This is evident also from the energy dependent ion yields in Figure 3. The spectra for isolated and hydrated conditions are very similar, only a slight increase of the parent ion signal at 2.8 eV is detected, caused by closing of the NO${}_{2}^{-}$ dissociation channel as demonstrated also for Nimorazole [30]. Furthermore, the dissociation via NO loss significantly decreases after low hydration, attaching of few (1–5) water molecules to the C${}_{4}$HCl${}_{2}$N${}_{3}$O${}_{2}$. On the other side, higher hydration does not have so pronounced effect, the formation of NO radicals may be expected even in bulk. This is also confirmed by our calculations, showing large exothermicity of the process with excess energy of 2.9 eV. So exothermic process may be initiated even by hydrated electrons, which are in bulk water bound in ∼1.5 eV potential well [60].

An important process, which we observe upon hydration is the nucleophilic substitution of chlorine by an OH group. We can see that even at low levels of hydration a new anion appears in the spectrum that can be asigned to electron attachment to C${}_{4}$H${}_{2}$ClN${}_{3}$O${}_{4}$ precursor. This reaction, even with low relative intensity in clusters, may play an important role in the bulk solutions. It is well known that the substitution reaction dictates the mode of cisplatin binding to DNA [61].

### 3.4. Cytotoxicity

Due to the cytotoxicity of the molecule, and therefore low viability of the cell cultures, it was not possible to further test its synergy with radiation. Additionally, the molecule has low solubility in water and biological buffers, suggesting that studies in a biological environment will require modification of the molecule. Here we provide details of the cytotoxicity study using MTT assays.

**Figure 4:**
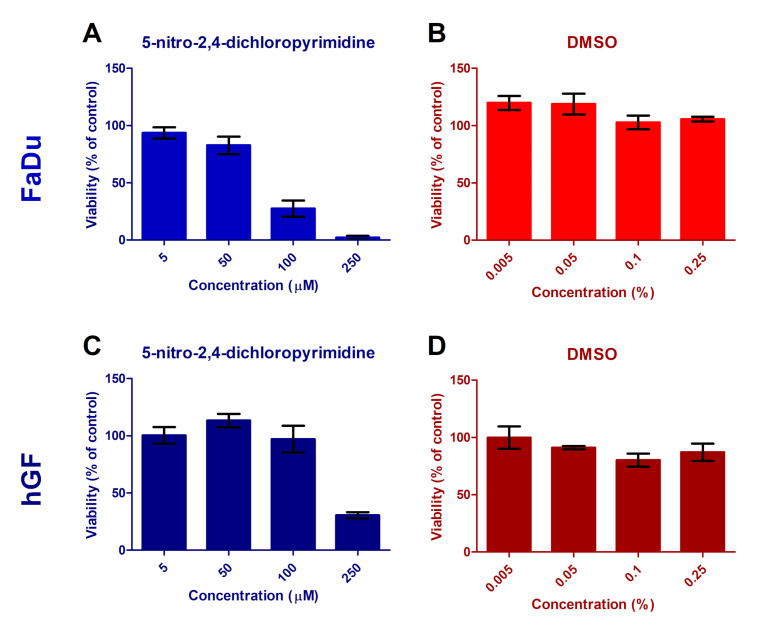
MTT assay evaluation of 5-nitro-2,4-dichloropyrimidine cytotoxicity for FaDu and hGF cell lines after 48 h incubation. Viability of cell lines treated by 5-nitro-2,4-dichloropyrimidine dissolved in DMSO (**A**,**C**) is shown as the % of corresponding DMSO-treated controls. Effect of corresponding concentration of DMSO (**B**,**D**) is shown as viability (%) compared with non-treated control. The results are plotted as the mean ± SEM values of 3-4 replicates performed in triplicates.

To test the cytotoxicity of the molecule we selected two unrelated permanent cell lines gingival fibroblasts (hGF) and epidermoid carcinoma (FaDu). hGF were selected as a model for untransformed cell lines that are irradiated during radiotherapy of many types of tumours. FaDu cells are then one of the most commonly used cell lines for basic oncology research. In Figure 4A,C, we can see toxicity of the molecule on the proposed cell lines. We can see that at concentrations above 100 μM the molecule is lethal for both cell lines. The toxicity seems to be slightly higher for FaDu cancer cell lines, which may be advantageous for the use of the molecule in cancer therapy. However, for similar, small electrophylic, radiosensitizers the combined effects with radiation were typically observed only at concentrations in mM range [7,62].

The molecule has low solubility in water and biological buffers and we reach reasonable dissolution only in dimethyl sulfoxide-DMSO. Even though DMSO is generally used as a non-toxic solvent there are several issues concerning its use in studies of radiation effects on cell lines that prevented our further exploration of the molecule. At first the effects of DMSO on cell lines range from proliferation enhancement at low concentrations [63,64] to toxicity at higher concentrations [65], which also depends on the cell line [66,67]. This is well demonstrated in Figure 4, panels B and D, showing cell viability in a buffer solution containing DMSO concentration equivalent to the DMSO content in the corresponding 5-nitro-2,4-dichloropyrimidine+DMSO experiments presented in Figure 4, panels A and C, respectively. We can see that DMSO has a positive proliferation effect on FaDu cells, while slightly negative effect on the hGF cells. Additionally the effects change at concentrations above 0.05%. These facts significantly complicate the dosing of the molecule solution for concentration dependent studies or cell line dependent studies. At the same time, DMSO is known as radioprotector [68]. However, DMSO radiation effects again strongly depends on the studied cell lines ranging from different pleiotropic effects [69] to no radio-protectivity at all [70]. The studies in a biological environment will therefore require modification of the molecule or completely new design of a molecule with higher biocompatibility.

## 4. Conclusions

We demonstrated that practically all known modes of action of low-energy electrons that has been hypothesised to cause synergy in concomitant chemo-radiation therapy may be combined in a simple small molecule.

We experimentally obtained parent anion lifetime, which is an important measure of the molecular stability upon electron attachment. The value of 1.2 ms is extremely long allowing further stabilization of the anion in cellular environment, multi electron reduction or effective transport of the anion through cellular membranes.

In combination with theory we estimated the electron affinity of the molecule in its isolated form and in the water environment to be above 2 eV. This energy is gained by the system by pure presence of the low-energy electrons that are formed in large amounts during radiation interaction with living tissue. High electron affinity may therefore enhance the LET value.

We also explored details of dissociative electron attachment and hydration reactions using clusters. We show that while the NO${}_{2}^{-}$ dissociation channel is suppressed in the water environment the Cl${}^{-}$ dissociation remains open.

Finally, we report nucleophilic substitution of Cl by OH group in microhydrated clusters, similar to the reaction of cisplatin in the bulk water environment. In the cellular environment this reaction may lead to better binding of the molecule to DNA.

However, we also demonstrate a high cytotoxicity of the molecule using MTT assays. This fact, together with low solubility of the molecule does not allow a reliable study of its combined effect with radiation on the cell viability.

We hope, the study will initiate more exploration of this topic and possible in vitro and in vivo studies of rationally designed molecules combining several modes of chemo-radiation synergism.

## Figures and Tables

**Table 1:** Assigned structures of fragment negative ions observed in DEA spectra, peak energies (eV) and relative intensities evaluated from the peak heights as obtained using the electron attachment spectrometer with sector mass analyzer. Thermodynamic thresholds for individual reaction channels are taken from DFT B3LYP/6-31G+(d) calculations in Gaussian [42].

m/z	Assigned Ion	Peak Energy (eV)	Relative Intensity (Max = 100)	Thermodynamic Threshold (eV)
193	**M${}^{-}$**	0	1.1	EAa = 2.122
177	[M – O]${}^{-}$	0	<0.1	
		2.7	0.1	1.336
163	**[M – NO]${}^{-}$**	0	100	−2.902
147	**[M – NO${}_{2}$]${}^{-}$**	0	75	−0.044
		2	4.9	
146	[M – HNO${}_{2}$]${}^{-}$	0	<0.1	
		2.5	<0.1	1.222
		4.1	<0.1	
128	[M – Cl – NO]${}^{-}$	0	0.2	0.965
127	[M – HCl– NO]${}^{-}$	0	<0.1	
		2.3	0.1	
		3.9	<0.1	
126	[M – Cl – O${}_{2}$]${}^{-}$	0	<0.1	
		2.7	0.1	
		8.5	<0.1	
122	[M – Cl${}_{2}$H]${}^{-}$	0	<0.1	
		2.6	0.1	2.425
111	[M – HCl – NO${}_{2}$]${}^{-}$	2.8	1.4	
99	C${}_{3}$N${}_{2}$Cl${}^{-}$	2.8	0.3	
93	[M – 2Cl – NO]${}^{-}$	2.9	0.1	
92	ClNCHNO${}^{-}$	3.2	0.1	
90	ClC${}_{2}$HNO${}^{-}$	2.8 sh.		
		4	<0.1	
		6–9 broad		
71	Cl${}_{2}$H${}^{-}$	0	<0.1	
		2.8	0.1	2.758
		3.9 sh.		
70	Cl${}_{2}^{-}$	2.9 sh.		2.471
		3.9	0.3	
		6–9 broad		
68	C${}_{2}$N${}_{2}$O${}^{-}$	0	<0.1	
		2.7	<0.1	
66	C${}_{3}$NO${}^{-}$	2.9		
		3.9 sh.		
64	C3N${}_{2}^{-}$	3.9	0.2	
	or ClN${}_{2}$H${}^{-}$	5.5 sh.		
		6–9 broad		
61	ClCN${}^{-}$	0	<0.1	
		3	0.1	
		3.9	0.1	
		6.7	<0.1	
50	C${}_{3}$N${}^{-}$	4	0.3	
		7–10 broad		
46	**NO${}_{2}^{-}$**	2.8	2.3	
		3.9 sh.		
		6.2 sh.		
42	OCN${}^{-}$	0	<0.1	
		3.7	0.1	
35	**Cl${}^{-}$**	0.15	5.4	
		2.7	22	
		3.8 sh.		
		5–9 broad		
26	CN${}^{-}$	0	<0.1	
		2.8	0.2	
		3.9	0.2	
		6.2 sh.		

**Table 2:** Electron affinities of the main decomposition products of 5-nitro-2,4-dichloropyrimidine, as obtained on DFT B3LYP/6-31+G(d) level of theory in Gaussian [42] and literature values in parentheses.

Neutral	AEA [eV]
M-NO	3.37
M-NO${}_{2}$	0.74
Cl	3.713 (3.614 [54])
NO2	2.33 (2.27 [55])
NO	0.87
